# Supplementary material for: Functionalization of 3D-Printed Titanium Scaffolds with Elastin-like Recombinamers to Improve Cell Colonization and Osteoinduction
Source: Pharmaceutics. 2023 Mar 8;15(3):872. doi: 10.3390/pharmaceutics15030872 (PMC10055514; doi:10.3390/pharmaceutics15030872)
Supplement: Supplementary file 1 [file pharmaceutics-15-00872-s001.zip › pharmaceutics-2158781-supplementary.pdf]

Supporting Information

Functionalization of 3D-printed titanium scaffolds with elastin-like recombinamers to improve cell colonization and osteoinduction

Jordi Guillem-Marti*, Elia Vidal, Alessandra Girotti, Aina Heras-Parets, Diego Torres, Francisco Javier Arias, Maria-Pau Ginebra, Jose Carlos Rodriguez-Cabello*, Jose Maria Manero.

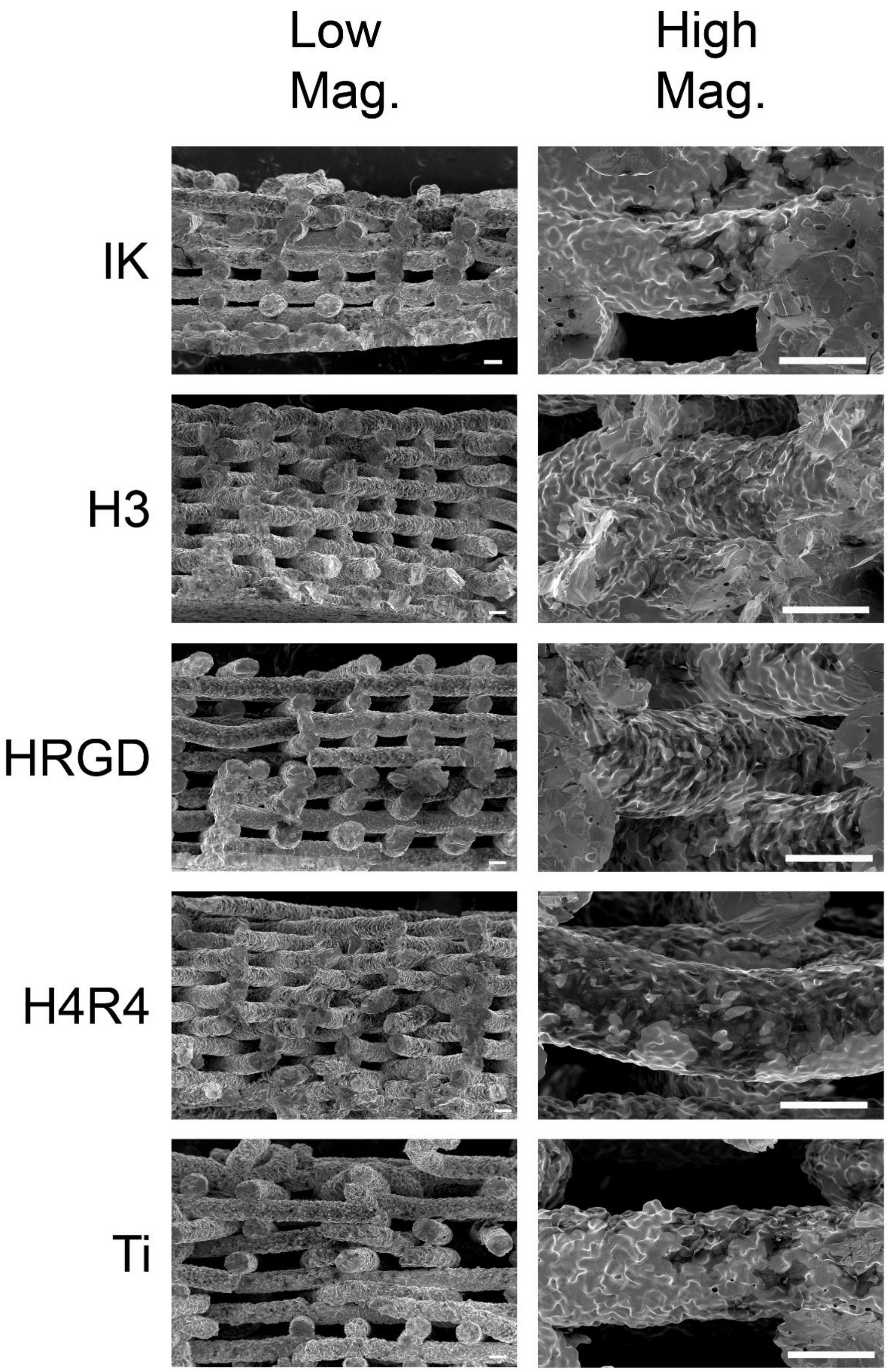

Figure S1. Low- and high-magnification SEM images of the different functionalized and non-functionalized Ti scaffolds showing attached cells 4 h after cell seeding. Scale bars denote 200 µm.

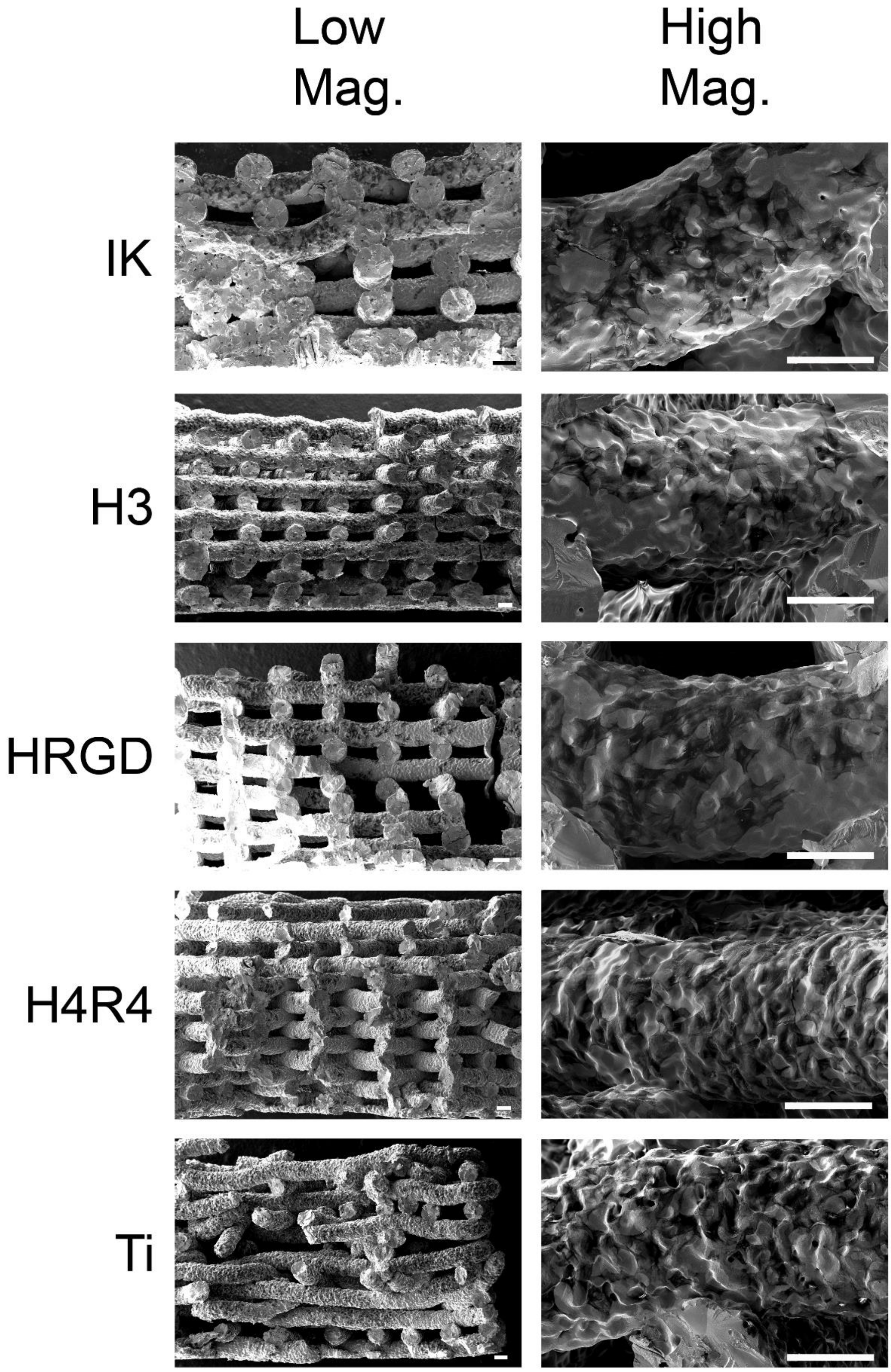

Figure S2. Low- and high-magnification SEM images of the different functionalized and non-functionalized Ti scaffolds showing attached cells after 7 days of culture. Scale bars denote 200 µm.

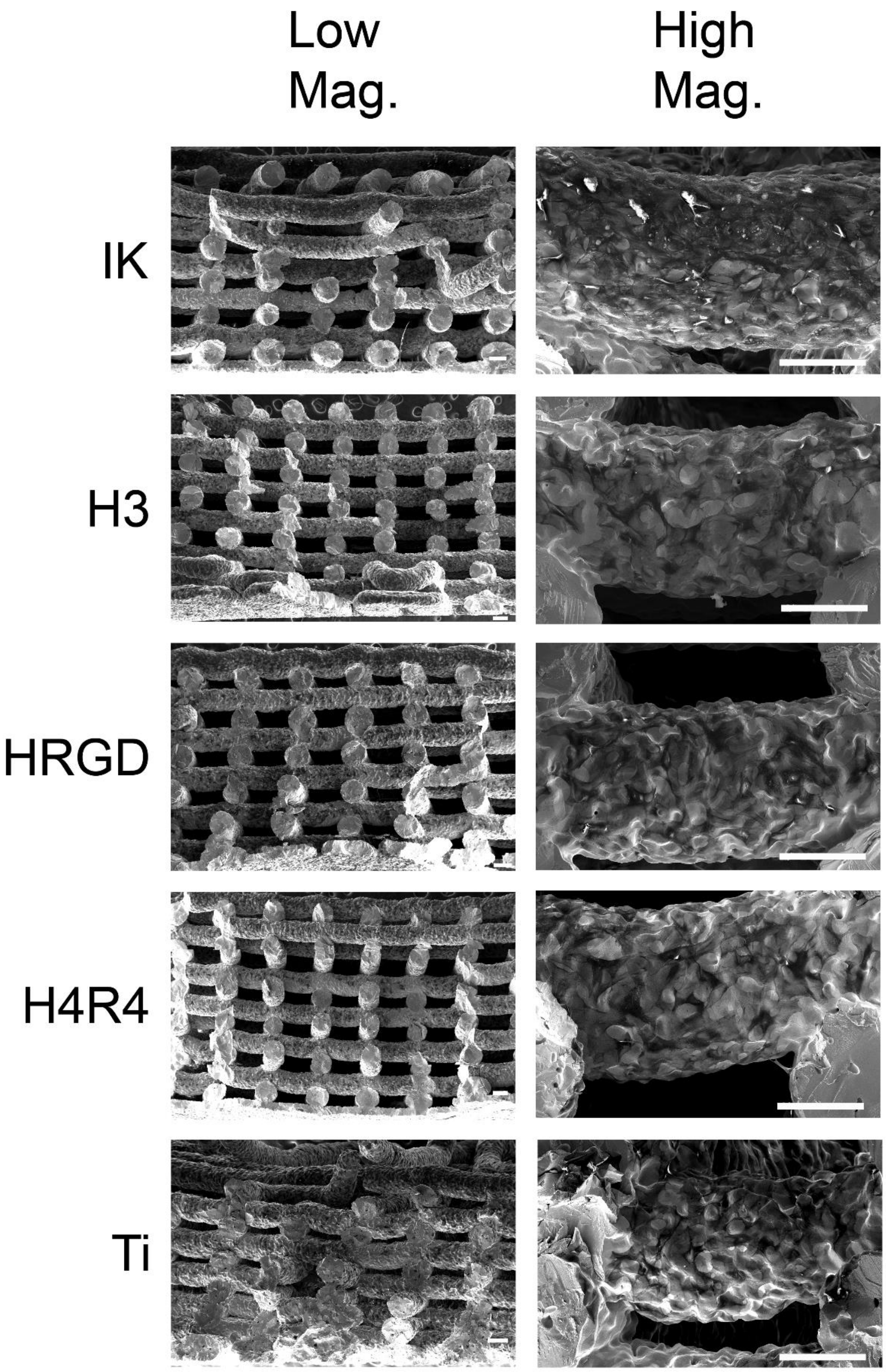

Figure S3. Low- and high-magnification SEM images of the different functionalized and non-functionalized Ti scaffolds showing attached cells after 14 days of culture. Scale bars denote 200 µm.

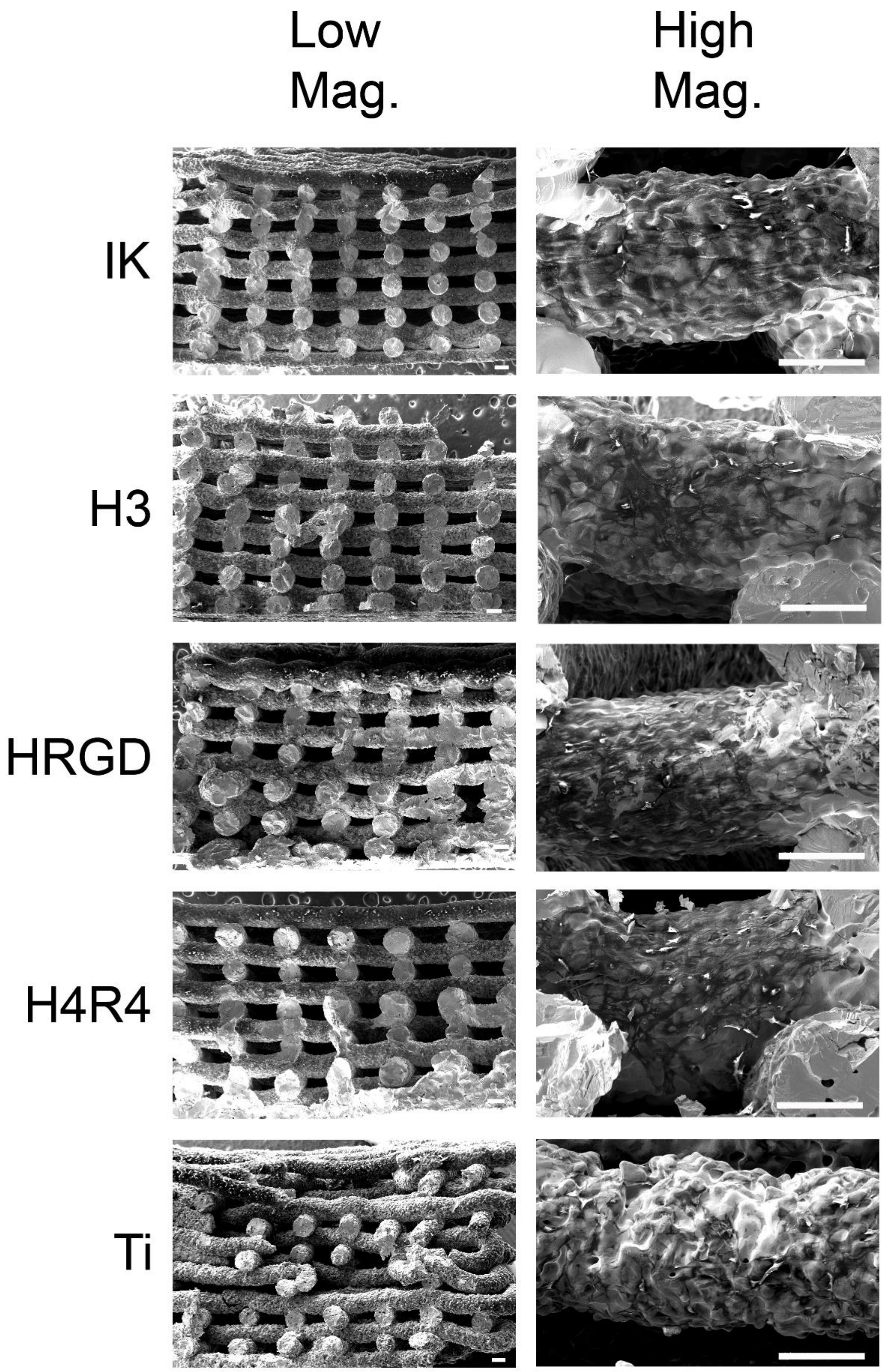

Figure S4. Low- and high-magnification SEM images of the different functionalized and non-functionalized Ti scaffolds showing attached cells after 21 days of culture. Scale bars denote 200 µm.

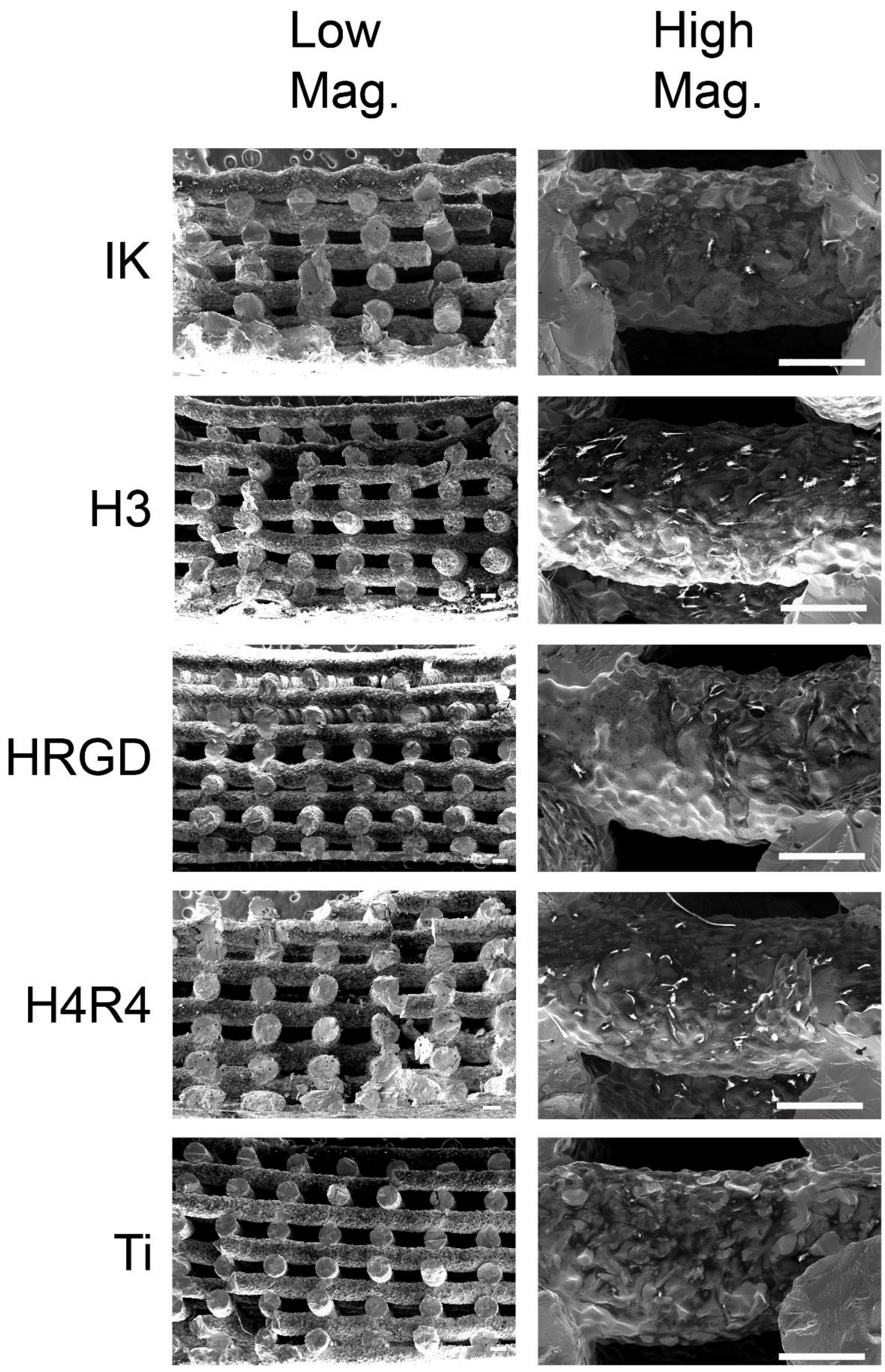

Figure S5. Low- and high-magnification SEM images of the different functionalized and non-functionalized Ti scaffolds showing attached cells after 28 days of culture. Scale bars denote 200 µm.
